# Automatic extraction of forward stroke volume using dynamic PET/CT: a dual-tracer and dual-scanner validation in patients with heart valve disease

**DOI:** 10.1186/s40658-015-0133-0

**Published:** 2015-10-26

**Authors:** Hendrik Johannes Harms, Lars Poulsen Tolbod, Nils Henrik Stubkjær Hansson, Tanja Kero, Lovisa Holm Orndahl, Won Yong Kim, Tomas Bjerner, Kirsten Bouchelouche, Henrik Wiggers, Jørgen Frøkiær, Jens Sörensen

**Affiliations:** Department of Nuclear Medicine & PET Centre, Aarhus University Hospital, Palle Juul-Jensens Boulevard 99, 8200 Aarhus N, Denmark; Department of Cardiology, Aarhus University Hospital, Palle Juul-Jensens Boulevard 99, 8200 Aarhus N, Denmark; Departments of Nuclear Medicine & PET, Uppsala University, Akademiska Sjukhuset, 751 85 Uppsala, Sweden; Department of Cardiology, Uppsala University, Akademiska Sjukhuset, 751 85 Uppsala, Sweden; Department of Radiology, Uppsala University, Akademiska Sjukhuset, 751 85 Uppsala, Sweden

**Keywords:** Positron emission tomography, Stroke volume, Dynamic imaging

## Abstract

**Background:**

The aim of this study was to develop and validate an automated method for extracting forward stroke volume (FSV) using indicator dilution theory directly from dynamic positron emission tomography (PET) studies for two different tracers and scanners.

**Methods:**

35 subjects underwent a dynamic ^11^C-acetate PET scan on a Siemens Biograph TruePoint-64 PET/CT (scanner I). In addition, 10 subjects underwent both dynamic ^15^O-water PET and ^11^C-acetate PET scans on a GE Discovery-ST PET/CT (scanner II). The left ventricular (LV)-aortic time-activity curve (TAC) was extracted automatically from PET data using cluster analysis. The first-pass peak was isolated by automatic extrapolation of the downslope of the TAC. FSV was calculated as the injected dose divided by the product of heart rate and the area under the curve of the first-pass peak. Gold standard FSV was measured using phase-contrast cardiovascular magnetic resonance (CMR).

**Results:**

FSV_PET_ correlated highly with FSV_CMR_ (*r* = 0.87, slope = 0.90 for scanner I, *r* = 0.87, slope = 1.65, and *r* = 0.85, slope = 1.69 for scanner II for ^15^O-water and ^11^C-acetate, respectively) although a systematic bias was observed for both scanners (*p* < 0.001 for all). FSV based on ^11^C-acetate and ^15^O-water correlated highly (*r* = 0.99, slope = 1.03) with no significant difference between FSV estimates (*p* = 0.14).

**Conclusions:**

FSV can be obtained automatically using dynamic PET/CT and cluster analysis. Results are almost identical for ^11^C-acetate and ^15^O-water. A scanner-dependent bias was observed, and a scanner calibration factor is required for multi-scanner studies. Generalization of the method to other tracers and scanners requires further validation.

## Background

The baseline definition of heart failure is the inability of the heart to pump sufficient amounts of blood to meet the metabolic needs of the organism or to do so only at an elevated filling pressure [1]. As the progression of almost any cardiac pathophysiology might evolve into heart failure, accurate measurements of stroke volume (SV) are fundamental in a clinical routine. Right heart catheterization is considered the golden standard but is used reluctantly due to its invasiveness. A great deal of effort has been put into qualifying the various clinically used cardiac imaging tests for stroke volume measurements. Cardiovascular magnetic resonance (CMR) with phase-contrast sequences has been established as the non-invasive golden standard for stroke volume measurements [2, 3].

Positron emission tomography (PET) is increasingly used for quantitative perfusion imaging in ischemic heart disease and functions as a gatekeeper for revascularization in patients with suspected coronary artery disease [4–6]. Widespread use is highly facilitated by standardized protocols and dedicated software for quantification [4, 7]. In addition, there is a growing interest in the use of PET to diagnose specific molecular and metabolic alterations both clinically and in research [8–10]. Some PET tracers with high myocardial retention lend themselves to ECG-gated assessments of left ventricular (LV) geometry and can thus be used for routine diagnosis of systolic LV derangement [11–13]. Other tracers, such as ^15^O-water and ^11^C-Pittsburgh compound B (^11^C-PIB), do not.

Access to reliable SV estimates directly from the dynamic PET data would enhance the value of many cardiac PET investigations. Similar to the invasive gold standard, these hemodynamic parameters are obtainable from PET by use of the indicator dilution principle, as previously shown [14]. Although the required data are easily acquired, the calculated parameters are rarely used with PET because of laborious manual procedures and lack of procedural standardization. The prerequisites are relatively simple: the left ventricular time-activity curve, the injected dose, and the heart rate. However, the intravenous bolus has to be rapidly and consistently delivered, the injected dose rigorously established, the heart rate recorded in a standardized fashion, and the blood pool time-activity curve manipulations have to account for shape variations. The aims of this study were to develop a systematic and fully automated approach for human SV measurements using any small-molecule PET tracer which can be applied routinely to any quantitative PET analyses and to evaluate the variation caused by the scanner device.

## Methods

### Patient population

This retrospective analysis included data of two patient cohorts undergoing both cardiac PET and CMR scans in the context of other studies. The first group, scanned on scanner I, consisted of 35 consecutive patients (25 men, age range 48.0–84.8 years, mean age 68.4 ± 9.6 years, 10 women, age range 52.6–86.5 years, mean age 67.9 ± 9.4 years) with aortic valve stenosis. This patient group was recruited and scanned at the Aarhus University Hospital, Aarhus, Denmark. All patients had sinus rhythm, no signs of myocardial ischemia and aortic valve area ≤1.2 cm^2^ ,and/or transaortic maximal velocity of 3.0–5.0 m/s based on echocardiography. The second group, scanned on scanner II, consisted of 10 consecutive patients with mitral regurgitation (10 men, age range 22–73 years, mean age 56.1 ± 15.9 years) and mild-moderate heart failure. This group was recruited and scanned at the Uppsala University, Uppsala, Sweden. All patients had significant (>20 %) mitral insufficiency according to echocardiography. The study was approved by the local ethical committees at both hospitals, and all patients gave written informed consent prior to inclusion in this study.

### Scanning protocol

#### PET

[^11^C]-acetate synthesis was done according to Pike [15] with minor in-house modifications.

The first patient group underwent a 27-min ^11^C-acetate PET scan on a Siemens Biograph TruePoint TrueV 64 PET/CT scanner (scanner I). Subjects were instructed to >4 h fasting prior to PET recordings, except for water and medicine prescribed for daily intake. After a scout CT scan, a low-dose CT scan (120 kV, 30 mAs, 4-mm slice thickness) was performed. Following this low-dose CT scan, a 27-min list mode emission scan was performed, starting simultaneously with injection of 400 MBq ^11^C-acetate as a 5- to 10-mL bolus (1 mL s^−1^) in a peripheral vein, using an automatic injection system, followed by a 35-mL saline flush (2.0 mL s^−1^) similarly as described in [16]. The list mode data were rebinned to give a dynamic series with 29 time frames (1 × 10 s, 12 × 5 s, 5 × 10 s, 2 × 30 s, 3 × 60 s, 3 × 120 s, 3 × 300 s) using all data. Dynamic images were reconstructed using the TrueX algorithm (3 iterations, 21 subsets, 5-mm 3D Gaussian post-filter) and routine corrections for attenuation, scatter, dead time, and decay as supplied by the vendor.

The second patient group underwent an ^15^O-water scan followed by a ^11^C-acetate scan on a GE discovery ST (scanner II). Acquisition protocol for ^11^C-acetate was identical to that of group I. For ^15^O-water, a 6-min list mode emission scan was performed, starting simultaneously with bolus injection of 400 MBq of ^15^O-water using the same injection protocol as described above. Emission data were acquired in list mode and reconstructed in a dynamic series with 22 time frames (1 × 10 s, 8 × 5 s, 4 × 20 s, 2 × 15 s, 3 × 20 s, 2 × 30 s, 2 × 60 s) for ^15^O-water. Data were reconstructed using the 3D IR algorithm (2 iterations, 21 subsets, 4.29-mm Gaussian post-filter) with routine corrections for attenuation, scatter, dead time, and decay as supplied by the vendor. To avoid contamination of the signal due to residual activity, the ^11^C-acetate scan was started at least 10 min (five half-lives) after the end of the ^15^O-water scan.

#### CMR

The first group of patients was scanned on an Ingenia 1.5 T whole-body scanner (Philips Healthcare, Best, The Netherlands). Based on three-chamber and left-ventricular outflow tract (LVOT) cine images, breath-hold through-plane phase-contrast CMR acquisitions were performed at the level of the LVOT. The following parameters were applied: echo time (TE) = 2.5 ms, repetition time (TR) = 4.1 ms, phase percentage = 60 %, field of view (FOV) = 350 mm, matrix = 140 × 140, number of phases = 25, number of excitations (NEX) = 1, and slice thickness (ST) = 8 mm. To avoid aliasing, encoding velocity was set to 100–200 cm/s based on pulse wave Doppler imaging from echocardiography performed just prior to CMR. To avoid biases and variability due to stenosis-induced velocity gradients or turbulence, imaging was performed at the level of the LVOT where flow is laminar.

The second group of patients was scanned on a ingenia 3 T whole-body scanner (Philips Healthcare, Best, The Netherlands). Scanning protocol was comparable to that of the first group of patients, with some differences. Based on orthogonal balanced turbo field echo images, respiratory triggered through-plane phase-contrast MR acquisitions were obtained at the level of the aorta ascendens during free breathing. Typical parameters were as follows: TE = 2.7 ms, TR = 4.7 ms, phase percentage = 81 %, FOV = 320 mm, matrix = 128 × 104, number of phases = 40, NEX = 2, ST = 8 mm, and encoding velocity = 100–200 kollas.

### Data analysis

The local standard method of calculating forward stroke volume using CMR (FSV_CMR_) differed slightly between the two centers. For the first group of patients, forward stroke volume (FSV)_CMR_ was calculated from phase-contrast velocity measurement in the LVOT using the freely available software Segment (version 1.9 R3746) [17]. For the second group of patients, FSV_CMR_ was calculated from phase-contrast velocity measurement in the aorta ascendens and flow analyses were performed on a ViewForum workstation (Philips, Best, the Netherlands).

**Fig. 1:**
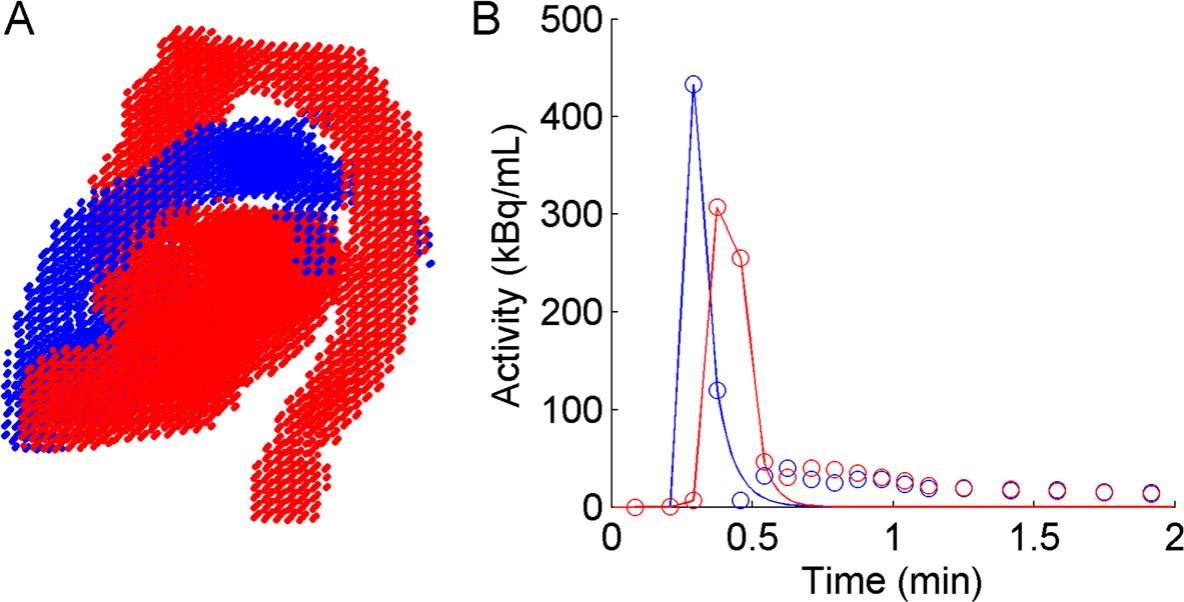
**a** Example of arterial (*red*) and venous (*blue*) clusters as obtained after cluster analysis. The arterial cluster includes the left atrium and ventricle and the aorta while the venous cluster includes the vena cava, right atrium and ventricle, and the pulmonary arteries. **b** Time-activity curves of the clusters in **a** for arterial (*red circles*) and venous (*blue circles*) blood and corresponding isolated first-pass peaks (*lines*)

FSV analysis of PET scans was implemented in Cardiac VUer [18], which is a software used by both centers for routine work and clinical research in cardiac PET. The software automatically obtains arterial and venous input functions using cluster analysis [18, 19]. These input functions are further used by the software for kinetic modeling, yielding fully quantitative myocardial blood flow values for ^15^O-water and rate of myocardial oxygen consumption for ^11^C-acetate. For ^15^O-water, six clusters were used while for ^11^C-acetate five clusters were found to yield more stable results. After visual inspection of the resulting clusters (Fig. 1a), the user selects the cluster representing the arterial blood pool (red in Fig. 1a) and the venous blood pool (blue in Fig. 1a) after which the average time-activity curve of each voxel in each cluster was used as final arterial and venous input functions. To reduce partial volume effects and spillover of activity from surrounding organs, the outer layer of voxels included in each cluster was eroded before input functions were obtained.

Then, FSV_PET_ was estimated fully automatically using the indicator dilution method [14]:

$$ \mathrm{F}\mathrm{S}{\mathrm{V}}_{\mathrm{PET}}=\frac{1}{\mathit{\int}{C}_A(t)\kern0.5em dt\times \mathrm{H}\mathrm{R}} $$

In which *FSV*_*PET*_ represents the forward stroke volume, *I* injected dose, *HR* the heart rate, and *C*_*A*_*(t)* the whole-blood time-activity curve (TAC) for the first-pass only. For calculation of FSV, the area under the curve of the first-pass peak of *C*_A_(*t*) was extracted fully automatically (Fig. 1b). First, the two successive frames *t*_1_ and *t*_2_ with a downslope greater than 0.75 times the maximum downslope were identified, and an exponential curve was fitted through these two frames. Then, the first-pass peak was defined as the activity of the original *C*_A_(*t*) or *C*_V_(*t*) up to frame *t*_2_ followed by the exponential fit from frame *t*_2_. The areas under the resulting curves were integrated by summing the products of time frame length and average activity for all time frames.

Correlation and agreement between FSV_CMR_ and FSV_PET_ was assessed using linear regression and Bland Altman plots, and a paired *t* test was used to assess the presence of systematic differences. Proportional errors were indicated by a significant correlation in Bland Altman plots while systematic errors were indicated by the mean difference between measurements. Repeatability coefficient (RPC) was calculated as two times the standard deviation (SD) of the differences between measurements or, in case of a proportional error, as two times SD of the residuals of the linear regression in the Bland Altman plot. FSV_PET_ was calculated using the arterial whole-blood time-activity curve (*C*_A_(*t*)) or the venous time-activity curve (*C*_V_(*t*)). In addition, the average of these values was obtained and compared to FSV_CMR_.

## Results and discussion

For one patient of scanner I, injected dose was not measured and two patients showed visually identifiable motion, and these patient had to be excluded from further analyses. Patient characteristics of the remaining patients are shown in Table 1. Hemodynamic parameters during PET are shown in Table 2. No significant differences in blood pressures and heart rates were found between ^15^O-water and ^11^C-acetate scans on scanner II. Blood pressures were comparable between patients scanned on scanner I and II while heart rate was significantly higher for the patients scanned on scanner I (*p* < 0.01).

**Table 1:** Patient characteristics in mean ± SD for continuous variables or N (%) for dichotomous variables

	Scanner I (*n* = 32)	Scanner II (*n* = 10)	*p* value
Male gender	23 (72)	10 (100)	0.001
Age (years)	68.9 ± 8.5	56.1 ± 15.9	0.033
BMI (kg/m^2^)	26.0 ± 3.7	24.5 ± 3.5	0.374
BSA (m^2^)	1.90 ± 0.18	2.04 ± 0.17	0.044
Hypertension	18 (56)	3 (30)	0.158
Diabetes	6 (19)	0 (0)	0.012
NYHA class			
I	19 (59)	7 (70)	0.556
II	8 (25)	2 (20)	0.75
III	4 (13)	1 (10)	0.833
Unknown	1 (3)	0 (0)	0.325
LVEF (%)	66 ± 11	60 ± 6	0.039
RF (%)	–	41 ± 11	–
Medication			
ACE or ATII inhibitors	9 (28)	3 (30)	0.915
Beta-blockers	4 (13)	2 (20)	0.616
Statins	20 (63)	0 (0)	<0.001
Diuretics	16 (50)	0 (0)	<0.001
Calcium antagonists	8 (25)	1 (10)	0.25

*BMI* body mass index, *BSA* body-surface area, *NYHA* New York Heart Association, *LVEF* left-ventricular ejection fraction, *RF* regurgitant fraction

**Table 2:** Blood pressures (in mmHg) and heart rate (min^−1^) of all patients groups

	SBP	DBP	MAP	HR
Scanner I 11C-acetate	138.2 ± 17.5	78.8 ± 10.3	98.6 ± 11.3	64.5 ± 11.5
Scanner II 11C-acetate	133.8 ± 17.4	72.3 ± 10.8	92.8 ± 11.9	56.8 ± 5.4**
Scanner II 15O-water	133.9 ± 16.4	72.1 ± 11.3	92.7 ± 12.1	57.1 ± 5.3**

*SBP* systolic blood pressure, *DBP* diastolic blood pressure, *MAP* mean arterial pressure, *HR* heart rate

**p < 0.01 vs scanner I ^11^C-acetate

Cluster analysis was performed automatically and successfully in all remaining patients. Analysis time was <1 min on a standard desktop PC. Average FSV values for both tracers and imaging modalities are shown in Table 3. A significant overestimation of FSV based on PET was found for all scanners and tracers, with the largest overestimation for scanner II.

**Table 3:** Average values for FSV (in mL) derived using CMR and PET

	CMR	Arterial blood	Venous blood	Average
Scanner I 11C-acetate	79.5 ± 19.6	98.9 ± 20.3*	99.4 ± 19.5*	99.1 ± 19.5*
Scanner II 11C-acetate	94.5 ± 17.6	139.1 ± 33.4*	132.9 ± 29.7*	136.0 ± 31.1*
Scanner II 15O-water	94.5 ± 17.6	136.3 ± 34.7*	143.6 ± 29.7*	140.0 ± 31.6*

*Denotes significantly different from CMR values (*p* < 0.001)

**Fig. 2:**
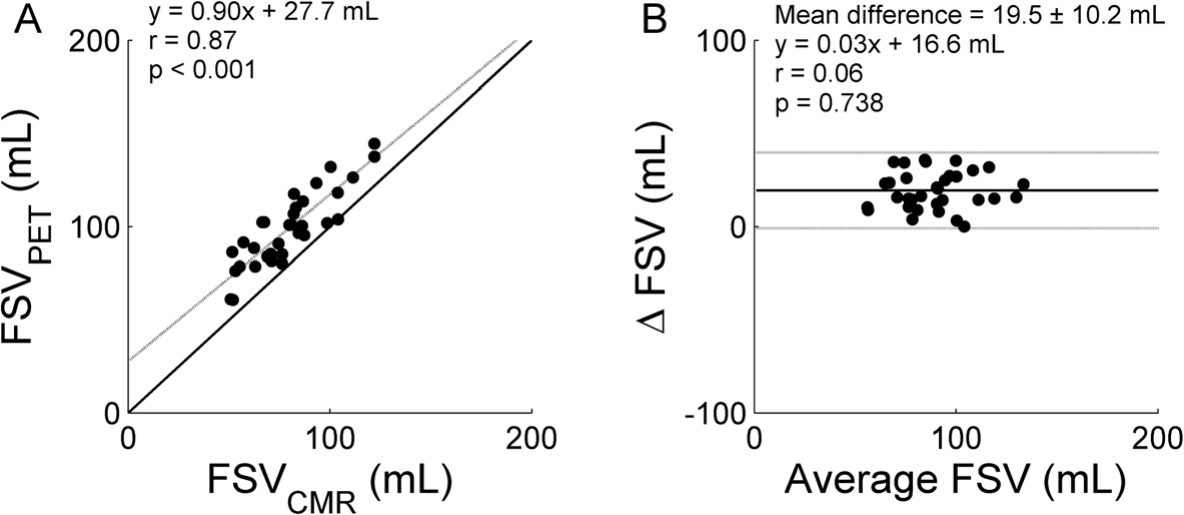
Correlation (**a**) between FSV_CMR_ and FSV_PET_ for scanner I based on ^11^C-acetate and arterial blood and its corresponding Bland Altman plot (**b**). Correlation coefficient, slope, and intercept of the linear fit were 0.87, 0.90, and 27.7 mL for FSV_PET_. No significant correlation was found in Bland Altman analyses. *Continuous lines* indicate the line of identity and *dotted lines* the linear fit in (**a**) and the mean difference and the 95 % confidence interval in (**b**)

**Fig. 3:**
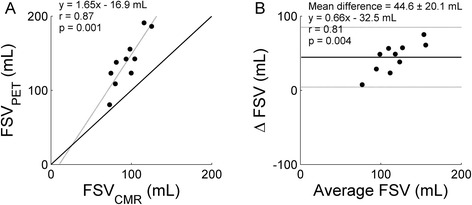
Correlation (**a**) between FSV_CMR_ and FSV_PET_ for scanner II based on ^11^C-acetate and arterial blood and its corresponding Bland Altman plot (**b**). Correlation coefficient, slope, and intercept of the linear fit were 0.87, 1.65, and −16.9 mL for FSV_PET_, and a significant correlation in Bland Altman analyses was found. *Continuous lines* indicate the line of identity and *dotted lines* the linear fit in (**a**) and the mean difference and the 95 % confidence interval in (**b**)

**Fig. 4:**
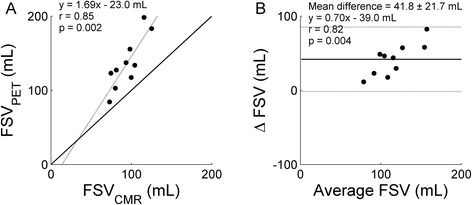
Correlation (**a**) between FSV_CMR_ and FSV_PET_ for scanner II based on ^15^O-water and arterial blood and its corresponding Bland Altman plot (**b**). Correlation coefficient, slope, and intercept of the linear fit were 0.85, 1.69, and −23.0 mL for FSV_PET_, and a significant correlation in Bland Altman analyses was found. *Continuous lines* indicate the line of identity and *dotted lines* the linear fit in (**a**) and the mean difference and the 95 % confidence interval in (**b**)

Figure 2 shows correlation between FSV_CMR_ and FSV_PET_ using arterial blood TACs for scanner I and its corresponding Bland Altman plot. A highly significant and high correlation was found when using arterial blood TACs (*r* = 0.87, *p* < 0.001). Similar but slightly lower correlations were found for venous blood TACs (*r* = 0.74, *p* < 0.001) or the average FSV_PET_ (*r* = 0.82, *p* < 0.001). Bland Altman analysis revealed a systematic error (*p* < 0.001) but no proportional error (*p* = 0.737). Correlation between FSV_CMR_ and FSV_PET_ for scanner II for ^11^C-acetate and ^15^O-water and their corresponding Bland Altman plots are shown in Figs. 3 and 4, respectively, based on the arterial blood TACs. Again, high and highly significant correlations were found when using ^11^C-acetate (*r* = 0.87, *p* = 0.001 for arterial blood; *r* = 0.86, *p* = 0.001 for venous blood; and *r* = 0.88, *p* < 0.001 for the average) and similar results were found when using ^15^O-water (*r* = 0.85, *p* = 0.002 for arterial blood; *r* = 0.88, *p* < 0.001 for venous blood; and *r* = 0.88, *p* < 0.001 for the average). Bland Altman analysis revealed both a systematic (*p* < 0.001) and proportional error (*p* = 0.004) for ^11^C-acetate and both a systematic (*p* < 0.001) and proportional error (*p* = 0.004) for ^15^O-water.

**Fig. 5:**
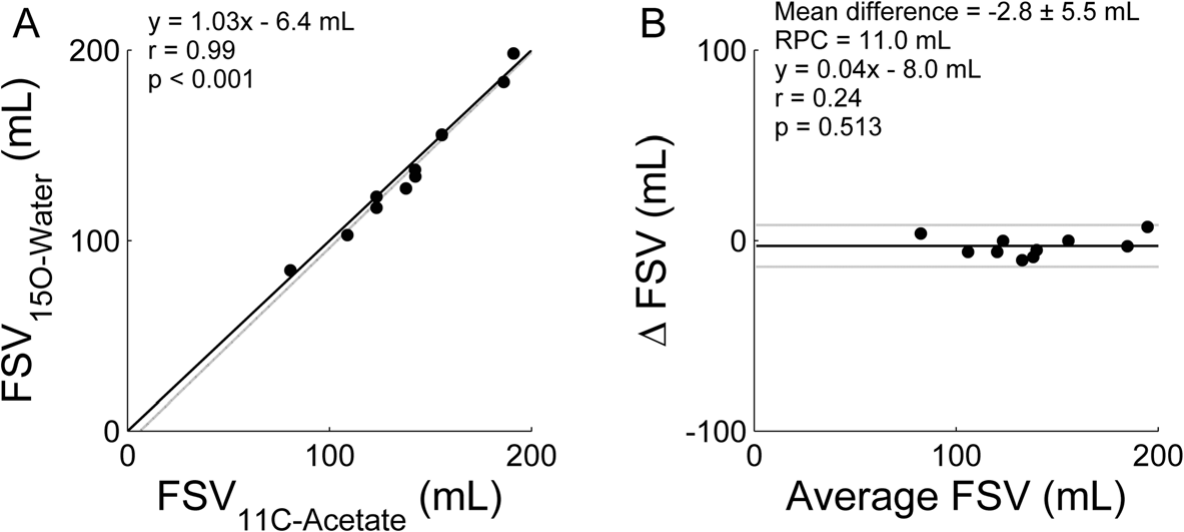
Correlation (**a**) between FSV_PET_ based on ^11^C-acetate and based on ^15^O-water when using arterial blood TACs and its corresponding Bland Altman plot (**b**). Correlation coefficient, slope, and intercept of the linear fit were 0.99, 1.03, and −6.4 mL, respectively, and no correlation was found in the Bland Altman plot. Repeatability coefficient was 11.0 mL. *Continuous lines* indicate the line of identity and *dotted lines* the linear fit in (**a**) and the mean difference and the 95 % confidence interval in (**b**). *RPC* repeatability coefficient

Correlation between FSV_PET_ based on ^11^C-acetate and ^15^O-water (Fig. 5) was close to unity (*r* = 0.99, *p* < 0.001) with no systematic (*p* = 0.14) or proportional (*p* = 0.513) difference between measurements. Repeatability coefficient for these measures was 11.0 mL.

### Discussion

This study shows the feasibility of a fully automated method of measuring forward stroke volume using the indicator-dilution principle and dynamic PET with two different tracers and scanners. The method requires no additional manual labor or separate PET reconstructions over those required for standard quantitative analysis of dynamic PET data. When using the arterial blood time-activity curve, a high correlation (*r* ≥ 0.85) was found with FSV as measured with the gold standard, phase-contrast CMR.

The method uses cluster analysis [18, 19] for extraction of image-derived input functions (i.e. *C*_A_(*t*)). This approach minimizes interobserver variability in quantitative analysis of myocardial blood flow [4, 18] and allows for integration of FSV_PET_ measurements in a clinical workflow without additional workload and independent of operator skill level. The method presented in this study is routinely applicable to any dynamic cardiac PET study, provided that a standardized and rapid infusion protocol is used and scan data with at least 90 s of short time frames is available.

There were no significant differences between the values obtained with ^15^O-water and ^11^C-acetate (*p* = 0.14). This illustrates the consistency of the method between different tracers and its high reproducibility. In addition, factors such as the increased positron range and shorter half-life of ^15^O or differences in uptake patterns between ^15^O-water and ^11^C-acetate did not affect the obtained results. This suggests that the method can be used with any tracer, as long as it is injected as a rapid bolus in a standardized fashion. In this study, automated injection devices were used in both centers and the infusion protocol was identical, eliminating possible biases due to differences in injection methodology. To reproduce the current results, care has to be taken to avoid a bolus injection that is too rapid as the low time-sampling typically found in PET studies limits the accuracy of boluses with too steep time-activity curves. Bolus infusion times of less than 5 s are therefore not recommended. In addition, care has to be taken that the infusion time is fast enough to minimize overlap between the first and second pass of the bolus through the blood pool. Including the second (or higher) pass in *C*_A_(*t*) will lead to overestimations of the area under the curve of the first-pass peak and consequently an underestimation of FSV_PET_.

On the other hand, a systematic bias with CMR was found for both scanners (*p* < 0.001), and this bias was different between scanners. Differences in for instance scatter corrections, detector material and crystal dimensions, or counting performance during the first-pass might induce scanner-dependent differences in obtained FSV values. In addition, the influence on partial volume effects is expected to be scanner dependent, manifesting itself in differences in contrast recovery. Furthermore, it has been shown that using reconstructions with corrections for the point-spread function (such as the TrueX reconstruction used for group I) yield higher contrast recovery as compared to standard iterative reconstructions [20]. Increased contrast recovery leads to increased areas under the peak of the first-pass and consequently less overestimation of FSV values. This might explain some of the differences in results between the scanners used in this study. Standardized corrections for partial volume effects using scanner-dependent recovery coefficients might reduce or even eliminate some of these issues. However, aortic diameter is not consistent between cardiac patients and using a fixed (a priori) recovery coefficient might introduce different biases in the data as these coefficients are object-size dependent. Since correlation with FSV based on CMR was high for both scanners, scanner-specific correction factors can be derived and used to get consistent values independent of scanner used. Alternatively, when this is not possible, FSV_PET_ can be used in a relative fashion when patients are scanned multiple times, as reproducibility of the method is high (Fig. 5).

Irrespective of these scanner differences, both scanners yielded a systematic overestimation of FSV. Underestimations of the total blood activity due to the partial volume effect (PVE), which is especially prominent in the descending aorta and to a lesser extent in the ascending aorta, lead to overestimations of FSV. To evaluate the impact of PVE, one additional erosion step was applied to the obtained clusters, keeping only the most central voxels which should suffer less from partial volume effects. For scanner I, no differences in FSV were found as compared to values obtained without additional erosion (*p* = 0.13), suggesting that partial volume effects were not significant. On the other hand, for scanner II, FSV values were significantly lower after an additional erosion (4.0 ± 1.6 %, *p* < 0.001), showing that partial volume effects still play a minor but significant role for this older scanner. Nevertheless, other factors such as scanner counting performance at high dead times, scatter corrections, and accuracy of the injected dose remain that might play a role and could be investigated further. However, the high correlation coefficients observed in this study show the consistency of the bias, and potentially correction factors can be applied for a more routine setting on current generation of PET/CT scanners. This however requires further validation.

Phase-contrast CMR is a versatile and thoroughly validated technique, at least theoretically not associated with scanner-related or operator bias although this has not been documented. The protocols might differ, as in this study, which potentially accounts for some differences in mean FSV_CMR_ between the two cohorts. In this study, there were three main differences in CMR protocol between sites. First, for patients investigated with scanner I, flow velocity was measured at the level of the LVOT because in patients with a stenotic aortic valve, flow is not laminar downstream of the aortic valve, and therefore flow measurements would inevitably be subjected to larger variation if the sampling site was chosen to be the ascending aorta. In patients investigated with scanner II, flow was measured in the ascending aorta. However, these patients did not have a stenotic aortic valve, flow was assumed to be laminar in the ascending aorta and consequently, the impact of this difference in CMR protocol is expected to be marginal. Second, patients investigated with scanner I had CMR during breathhold, while patients in the second cohort had CMR during free breathing. Cardiac loading conditions change with the breathing cycle, and, as a consequence, the measured stroke volume differs. All PET scans were done with free breathing, and the bolus travels through either chamber during a few breaths. The excellent repeatability of the PET measurements performed with scanner II suggests that free breathing during first-pass PET does not induce significant errors. However, if PET is calibrated to CMR for the sake of measurement portability, the actual CMR protocol might affect the PET values. Finally, the temporal resolution was higher in patients scanned on scanner II (32 cardiac phases per beat) as compared to scanner I (15 cardiac phases per beat). For this reason, it is expected that FSV_CMR_ estimates are more precise and accurate for the former group although systematic differences are not expected. Finally, it has to be noted that differences in age, body-surface area, heart rate, loading conditions, and disease state between both groups may account for some differences in average FSV.

FSV measurements of the patients scanned on scanner II had a more pronounced bias, compared to CMR. These patients had significant mitral regurgitation, potentially enhancing the bias. Phase-contrast CMR separates outward and backward flow velocities while the PET method only measures net outflow of the bolus. The PET application of FSV measurements is an implementation of standard indicator dilution technique, similar to the invasive thermodilution approach, and also successfully applied for decades using radionuclides and external gamma counters [21]. PET has a much lower time resolution than both temperature probes and gamma cameras, but since the clusters (Fig. 1) incorporate both the left-ventricular and the left-atrial cavity, regurgitated blood is still included in the arterial cluster. This will increase the area under the first-pass curve proportionally, and mitral regurgitation is therefore accounted for when estimating FSV_PET_. This was confirmed by the excellent correlation between FSV_CMR_ and FSV_PET,_ as well as the excellent correlations between FSV_PET_ for both venous and arterial blood. Consequently, the bias observed with scanner II is related to the PET device and not the cardiac condition or the indicator dilution principle per se.

The method applied in this study utilizes cluster analysis; an automatic segmentation method which is use in cardiac PET has been shown before [18] and is successfully applied in a clinical setting [4]. This is in contrast to other studies using manually defined blood TACs [14, 22, 23] or semi-automated methods [24, 25]. The automated segmentation method has the obvious advantage of reducing or eliminating observer-induced variation and increasing workflow. Since arterial input functions are fundamental for any quantitative PET study, the method described in this study requires no extra work other than entering the heart rate during the start of the scan and the injected dose. The proposed method is therefore easily applied to a clinical routine. In addition, isolation of the first-pass peak from *C*_A_(*t*) or *C*_V_(*t*) is performed fully automatically. In contrast to [23], no assumptions are made regarding the upslope of the peak, and except for the exponential fit starting after the frame with the maximum downslope (*t*_2_), the original data of *C*_A_(*t*) and *C*_V_(*t*) are retained. Fewer processing steps to the data are likely to limit potential biases and errors due to post-processing.

There are several limitations to the method described in this study. First, the injected dose must be administered as a rapid bolus in order to accurately isolate the peak of the first-pass. In cases where the bolus is fractionated or where a significant part of the bolus is for instance stuck in the arms, the relationship between the injected dose and the area of the first-pass peak (Eq. 1) is compromised and results are unreliable. Similarly, when the injection is performed as a slow bolus or an infusion, as is typically the case when using ^82^Sr/^82^Rb generators, overlap between the first and second pass of the tracer can occur and will influence obtained values of FSV. Consistent tracer administration methods are therefore required for accurate measurements of FSV. The patients included in this study all had valvular abnormalities. Because of this, a comparison between regular stroke volume based on CMR or ECG-gated PET data and FSV_PET_ was not performed, as significant differences between total stroke volume and forward stroke volume cannot be ruled out.

Left-ventricular ejection fraction (LVEF) may be a more powerful predictor of cardiac events than SV alone. However, LVEF requires not only SV but also end-diastolic volume which cannot obtained using the methods described in this study, ruling out calculation of LVEF. More studies are warranted for automated and routine extraction of end-diastolic volume from dynamic PET images, enabling subsequent calculation of LVEF.

This study shows the validity of FSV_PET_ for ^11^C-acetate and ^15^O-water. In spite of the highly different downstream distribution pattern, these tracers performed equally well for measuring FSV. This suggests that the new method is directly applicable to most other and more widely used tracers, such as ^18^F-FDG and ^13^N-ammonia. ^82^Rb-rubidium generators may have variable infusion schemes based on generator age, which will require additional validation of FSV measurements in that setting. Similarly, additional validation is also needed for tracers with potentially very high first-pass lung retention, such as ^11^C-PIB in studies of cardiac amyloidosis and some tracers of highly lipophilic drugs. However, for some of these tracers, absolute quantification is not routinely performed, and static uptake images are used instead, often accompanied with ECG-gated reconstructions. Performing separate dynamic reconstructions to obtain FSV_PET_ has little value over the use of regular SV based on ECG-gated reconstructions in these cases. The use of FSV_PET_ can therefore only be recommended for tracers which frequently require dynamic scanning and absolute quantification, as the method can be incorporated in the standard analysis only in these cases.

## Conclusions

Forward stroke volume can be obtained automatically and reliably using dynamic PET/CT and cluster analysis. Although a scanner-dependent bias was found, the method was independent of the tracer used in this study and can in theory be extended to other tracers. This allows automated, rapid, and routine calculation of forward stroke volume with any dynamic cardiac PET examination.

## Compliance with ethical standards

All procedures performed in studies involving human participants were in accordance with the ethical standards of the institutional and/or national research committee and with the 1964 Helsinki declaration and its later amendments or comparable ethical standards. This article does not contain any studies with animals performed by any of the authors.

The study was approved by the local ethical committees at both hospitals, and all patients gave written informed consent prior to inclusion in this study.
